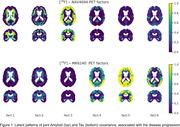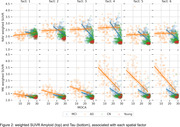# In vivo data‐driven patterns of Amyloid‐ and Tau accumulation associated with AD progression using 18F‐MK6240 and 18F‐NAV4694 PET

**DOI:** 10.1002/alz.094000

**Published:** 2025-01-09

**Authors:** Vladimir S Fonov, Pedro Rosa‐Neto, D Louis Collins

**Affiliations:** ^1^ McConnell Brain Imaging Centre, Montreal Neurological Institute, McGill University, Montreal, QC Canada; ^2^ Translational Neuroimaging Laboratory, The McGill University Research Centre for Studies in Aging, Montréal, QC Canada

## Abstract

**Background:**

Alzheimer’s disease is a neurodegenerative disease associated with the accumulation of Amyloid‐ß and Tau neurofibrillary tangles following a pattern known as Thal and Braak stages, respectively (Thal 2002; Braak 1995,2011). Recent research (Pascoal 2020) showed the possibility of recapitulating Braak’s histopathological stages in vivo using PET tracer [18F]‐MK‐6240 with manually defined regions of interest. This study analyzes the joint patterns of Amyloid‐ß and Tau accumulation associated with AD in a completely data‐driven fashion.

**Method:**

We used T1w MRI, [18F]‐NAV4694, and [18F]MK6240 PET scans from 36 young individuals (47 scans), 192 healthy elderly individuals (280 scans) in the community or outpatients at the McGill University Research Centre for Studies in Aging (97 individuals with mild cognitive impairment (140 scans) and 75 patients with AD (98 scans). T1w MRIs were pre‐processed (non‐uniformity correction, intensity normalization, stereotaxic registration, brain masking, tissue classification) and non‐linearly registered to the ADNI template using ANTs (Avants et al 2011). PET scans were linearly registered to the T1w MRI, and voxel‐wise standardized uptake value ratios (SUVR) were calculated using whole cerebellum grey matter as the reference, and then non‐linearly warped into the space of the ADNI template and downsampled to 2mm3 resolution. All PET scans were concatenated to create a matrix 518×345846. This matrix was factorized into two low‐rank components (Krichene 2018) with the constraint that all matrix elements should be positive, spatial loadings constrained to be between 0 and 1, latent vectors ordered monotonously to capture the additive nature of disease progression, and the loadings aligned with MOCA scores, by minimizing cosine distance. We used spatial components to create pseudo‐probabilistic ROIs associated with the different stages of disease progression.

**Result:**

Our method revealed regional patterns of joint tau and amyloid accumulation associated with AD progression, shown in Figure 1. Associated weighted SUVR average values are shown on Figure 2. The first two components, as visible on Figure 2, have almost no relationship with MOCA score and are most likely associated with off‐target bindings for both tracers.

**Conclusion:**

This work is a stepping stone towards creation of the Tau & Amyloid AD disease staging system.